# Assessing awareness of colorectal cancer symptoms, risk factors and screening barriers among eligible adults in Jordan: a cross-sectional study

**DOI:** 10.1186/s12889-025-22800-6

**Published:** 2025-04-25

**Authors:** M. S. El Muhtaseb, Aseel Ghanayem, Wa’ad N. Almanaseer, Husam Alshebelat, Rawan Ghanayem, Ghadeer M. Alsheikh, Fahed Al Karmi, Daoud O. Al Aruri

**Affiliations:** 1 Department of General Surgery, School of Medicine, University of Jordan, Amman, Jordan; 2 School of Medicine, University of Jordan, Amman, Jordan; 3 School of pharmacy, University of Jordan, Amman, Jordan; 4 Department of General Surgery, Jordan University Hospital, University of Jordan, Amman, Jordan

**Keywords:** Colorectal cancer (CRC), Awareness, Risk factors, Screening, Barriers

## Abstract

**Background:**

Colorectal cancer (CRC) is the second most prevalent malignancy in Jordan. Because early detection can greatly improve treatment outcomes, it is crucial to increase awareness of signs and symptoms, risk factors, and the significance of routine CRC screenings. In this study, we aimed to assess awareness levels regarding CRC symptoms and risk factors among adults in Jordan and to identify barriers to CRC screening.

**Methods:**

This web-based cross-sectional study was conducted in Jordan from March 5, 2024 to July 9, 2024, and targeted people aged 50–75 years who had no history of CRC. The sample size was calculated via a convenience sampling method. Data were collected via a validated, culturally adapted survey. Descriptive analysis was used when appropriate. Analytic statistics were performed to predict participants’ awareness of CRC symptoms and risk factors.

**Results:**

The study included 400 participants, with a mean age of 58.42 years (SD = 6.511). More than half of the respondents were females (56.5%). The mean awareness score of CRC symptoms among the study participants was 4.97/9 (SD = 1.18), whereas that of risk factors was 5.21/10 (SD = 1.53). The overall mean awareness score was 10.18/19 (SD = 2.65). The top three reported barriers to CRC screening were: not at risk due to absence of symptoms (61.8%), not at risk due to adopting a healthy lifestyle (56.8%), not at risk due to absence of family history (51.8%).

**Conclusion:**

Colorectal cancer awareness among the population was relatively low, with significant symptoms and risk factors being overlooked by the participants. In addition to that, notable barriers to screening, especially fear and embarrassment of the screening test, have surfaced. This prompts the need for more cancer education and healthcare provider involvement to overcome screening barriers and promote participation in screening programs to enable early detection.

**Supplementary Information:**

The online version contains supplementary material available at 10.1186/s12889-025-22800-6.

## Background

Colorectal cancer (CRC) is a major public health concern, ranking as the third most common cancer worldwide and the second leading cause of cancer-related mortality [1]. With an estimated 52,550 deaths predicted in 2023, it ranks as the second most common cause of cancer-related deaths in the United States for both men and women [2]. It is anticipated that the number of CRC cases would increase by 63% by the year 2040, leading to around 3.2 million new diagnoses and 1.6 million deaths annually [3]. In Jordan, CRC ranks as the second most commonly diagnosed cancer [4]. According to GLOBOCAN 2022, CRC constitutes 16.5% of all cancer cases in Jordan, ranking among the top three causes of cancer-related mortality for both men and women, following lung and breast cancers [1].

CRC primarily affects those aged 50 years and older [3], which led to the recommendation of the United States Preventive Services Task Force (USPSTF) to start screening for CRC at age 50 [5]. The USPSTF standards have been followed by Jordan’s healthcare authorities in regard to the adoption of CRC screening. Recently, the USPSTF updated its guidelines to include individuals aged 45 to 49 years in CRC screening [5]. This change marks a significant advancement in reducing CRC morbidity and mortality by enabling earlier diagnosis and treatment.

Although CRC is very preventable and curable if caught early, it remains a serious public health challenge, especially in regions with low screening rates [6]. It has been demonstrated that early identification through screening lowers morbidity and mortality [7]. Nonetheless, there is still a lack of knowledge on CRC symptoms, which include altered bowel habits, rectal bleeding, and unexplained weight loss. It is also essential to understand the risk factors associated with CRC, such as age, family history, and lifestyle choices, in order to promote screening program participation and preventative behaviors [8].

While healthcare organizations in Jordan have adopted screening standards consistent with global recommendations, the country still lacks a comprehensive national CRC screening program. This is due to an apparent lack of data regarding barriers and obstacles that impede individuals from engaging in routine screening, as only a few studies have explored these challenges in depth [9, 10, 11]. These barriers may include lack of knowledge about CRC, fear of the procedure, cultural stigmas, limited access to healthcare services, and financial constraints [12, 13]. Addressing these challenges is critical to increasing screening rates and reducing CRC-related morbidity and mortality [14].

We hypothesize that awareness of CRC symptoms and risk factors is low among older individuals in Jordan. Thus, the primary aim of this study was to provide a thorough assessment of public awareness of CRC symptoms and risk factors among individuals aged 50–75 years in Jordan. The secondary aim was to explore the obstacles that prevent CRC screening in the population. By focusing on these areas, we aim to enhance overall health outcomes and reduce the incidence of CRC in Jordan by raising awareness, minimizing screening barriers, and providing insights to help design an effective CRC screening program.

## Materials and methods

### Design

**Fig. 1:**
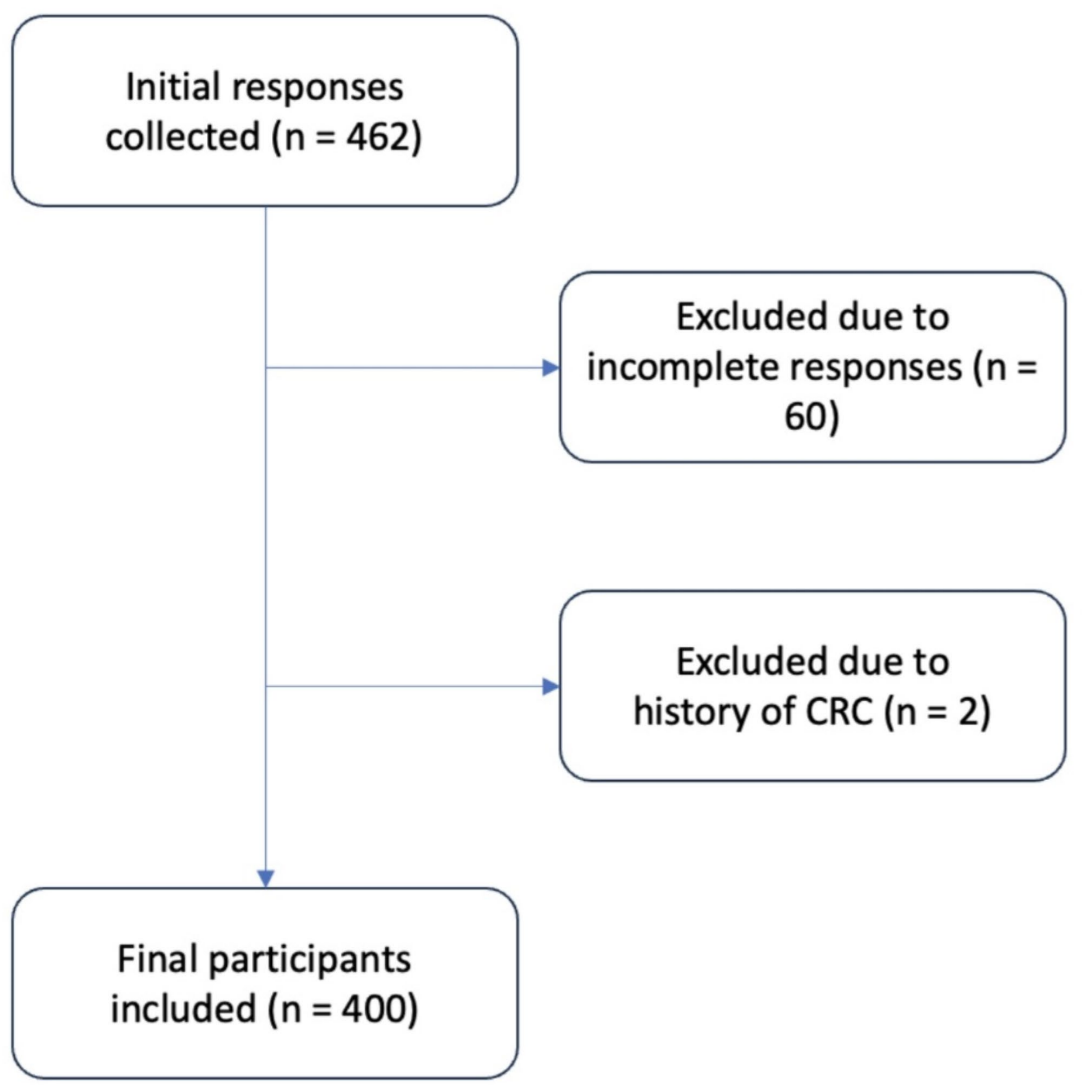
Flow chart of the inclusion and exclusion criteria for participants

We conducted a web-based cross-sectional study in Jordan between March 5, 2024, and July 9, 2024, using a validated questionnaire (Additional file 1). A cross-sectional design allows for capturing the current awareness level among the population, which helps in tailoring an immediate public health intervention. The eligible population included individuals aged 50–75 years with no prior history of CRC and who were able to complete the questionnaire in Arabic. Participants were excluded if they met any of the following criteria: [1] aged below 50 or above 75 years [2], a personal history of CRC [3], incomplete questionnaire responses [4], occupation as medical personnel, or [5] participants who had previously completed the survey. However, no participants were excluded based on age criteria, and no responses were received from medical staff. Also, none of the participants completed the survey more than one time. Therefore, the final sample consisted of non-medical respondents within the eligible age range who completed the questionnaire in full. (Fig. 1).

### Sample size

We adopted the convenience sampling method in our study. The Raosoft sample size calculator was used to estimate the sample size [15]. With a 5% margin of error, a 95% confidence level, and a 50% response distribution, the sample size was calculated to be at least 385 participants.

A 50% response distribution was chosen because it represents the most conservative estimate, maximizing the required sample size. This assumption was made due to the limited available information regarding the expected response patterns in our target population.

### Questionnaire

Our questionnaire covered two main aspects: CRC awareness and barriers to screening. The awareness part was adopted from the Bowel/Colorectal Cancer Awareness Measure (Bowel/Colorectal CAM) survey, which provides a validated questionnaire that was developed by the University College London and Cancer Research UK [16]. The original questionnaire was reviewed by a colorectal surgeon and a public health expert to ensure cultural appropriateness. After that, it was translated and back translated by bilingual experts to ensure clarity. Regarding barriers to screening, they were evaluated using a pre-existing questionnaire by a study that was conducted in Qatar [17]. Our data collection tool demonstrated excellent overall reliability and internal consistency (Cronbach’s alpha = 0.832). Subscale reliabilities for each part of the questionnaire were also good. Questions about CRC symptoms, risk factors, and barriers to screening achieved a Cronbach’s alpha of 0.783, 0.719, and 0.769, respectively.

The study’s questionnaire consisted of four main sections: participants’ sociodemographic characteristics (Sect. 1), knowledge about CRC signs and symptoms (Sect. 2), risk factors (Sect. 3), and barriers to the early screening program (Sect. 4).

Section 1 included six questions regarding background characteristics, which were age, sex, nationality, marital status, education level, and employment status. Section 2 featured nine closed-ended questions assessing participants’ awareness of CRC symptoms, while Sect. 3 contained ten closed-ended questions related to participants’ awareness of CRC risk factors. The response options for Sects. 2 and 3 were: yes, no, and do not know. Section 4 included 11 questions addressing barriers to early screening programs, where participants responded to these closed-ended questions using the following options: do not know, strongly disagree, somewhat disagree, neutral, somewhat agree, and strongly agree.

Sections 2 and 3 were used to assess the participants’ awareness of CRC symptoms and risk factors. The total score ranged from 0 to 9 for Sects. 2 and 0 to 10 for Sect. 3, and by combining them, we reach a total score of 19. The entire questionnaire was pilot-tested among 25 participants from the study population through face-face interviews before full deployment, to ensure clarity and reliability. Participants in pilot testing group reported no significant challenges in understanding the questions, and all items were answered without difficulty. Additionally, there were no reports of survey fatigue, indicating that the questionnaire length was appropriate. Based on these findings, the questionnaire was considered valid and suitable for use in the main study without requiring further modifications.

### Data collection

A web-based Google Form was distributed through social media platforms for data collection. The data were recorded anonymously without any contact or personal information. At the beginning of the questionnaire, participants were given the option to consent or decline participation in the study. If they chose to participate, they were presented with four confirmatory questions. The first question ensured that they had not previously completed the questionnaire for the same study, preventing data duplication. The second question verified the participants’ eligibility based on age. The third question determined their eligibility based on whether they had a personal history of CRC or not, and the fourth question confirmed that they were not medical personnel, ensuring that only non-medical respondents were included in the study.

Participants with incomplete responses, those under the age of 50 or over the age of 75, and those with a history of CRC were excluded to prevent potential information bias. Initially, we had 462 responses, but after applying the exclusion criteria, we were left with 400 responses. Data from the online questionnaire were automatically collected and stored in an Excel spreadsheet. Responses in Arabic were translated into English within the same spreadsheet for analysis.

Our data collection tool may be subject to biases such as recall bias and social desirability. To minimize their effect on the results, the questions were designed to be simple and straightforward, which reduces self-reporting bias, and the anonymity of the participants encouraged more honest answers.

### Data analysis

Our statistical analysis was performed using Statistical Package for the Social Sciences (SPSS) V.23. The participants’ responses to the questions assessing awareness of symptoms and risk factors were converted into binary variables (1 for correct, 0 for incorrect). Frequencies and percentages were then used to summarize the categorical variables, while means and standard deviations were calculated for continuous variables. We examined the variation in awareness mean scores across the different groups in the sample using Analysis of Variance (ANOVA) test. Chi-square test was employed to assess the association between categorical variables. To identify predictors of CRC awareness, univariate and multiple linear regression analyses were performed, with results expressed as mean differences and 95% confidence intervals. The level of statistical significance was set at 0.05. Regarding screening barriers questions, responses were reported using frequencies and percentages.

The participants’ responses to the questions assessing awareness of symptoms and risk factors were converted into binary variables (1 for correct, 0 for incorrect). Answers ‘’yes’’ were given ‘’1’’, and ‘’No’’ and ‘’Do not know’’ were given ‘’0’’. To assess the association between demographic characteristics and barriers to CRC screening, answers ‘’Strongly agree’’ and ‘’Somewhat agree’’ were given ‘’1’’, while ‘’Do not know’’, ‘’strongly disagree’’, ‘’somewhat disagree’’, and ‘’Neutral’’ were given ‘’0’’. Converting data into binary variables enabled a straightforward comparison of awareness levels across different demographic groups and facilitated the calculation of mean awareness scores. Additionally, it allowed for the examination of associations between various demographic characteristics and barriers to CRC screening.

## Results

### Demographic characteristics

Four hundred individuals participated in the study. Table 1 presents the participants’ background characteristics. The participants’ mean age was 58.42 years (SD = 6.511). The majority of the respondents were female (56.5%), Jordanian (96.8%), married (85.8%), and university educated (66.5%), with nearly half being retired (46%).

**Table 1:** Background characteristics of the participants (*N* = 400)

Variable	Frequency (*n*)		Percent (%)
Age			
50–59	245	61.3	
60 or more	155	38.8	
Sex			
male	174	43.5	
female	226	56.5	
Nationality			
Jordanian	387	96.8	
Non-Jordanian	13	3.3	
Marital Status			
single	57	14.3	
married	343	85.8	
Educational Level			
Pre-secondary	49	12.3	
secondary	85	21.3	
university	266	66.5	
Occupational Status			
employed	89	22.3	
self-employed	33	8.3	
unemployed	94	23.5	
retired	184	46.0	

### Awareness of CRC symptoms and risk factors

The mean awareness score of CRC symptoms among the study participants was 4.97/9 (SD = 1.18), while that of risk factors was 5.21/10 (SD = 1.53). The overall mean awareness score was 10.18/19 (SD = 2.65).

**Fig. 2:**
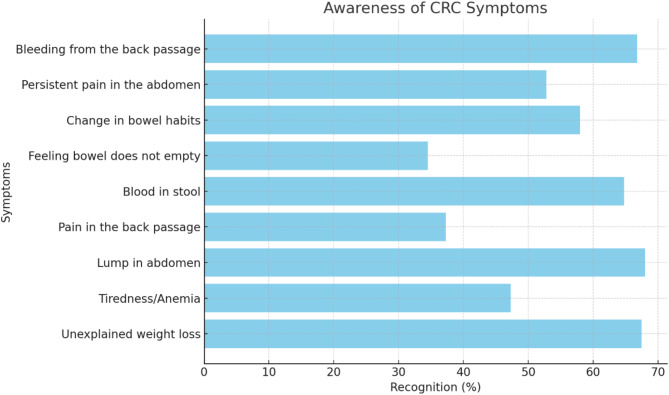
The recognition percentages of CRC symptoms among participants

**Fig. 3:**
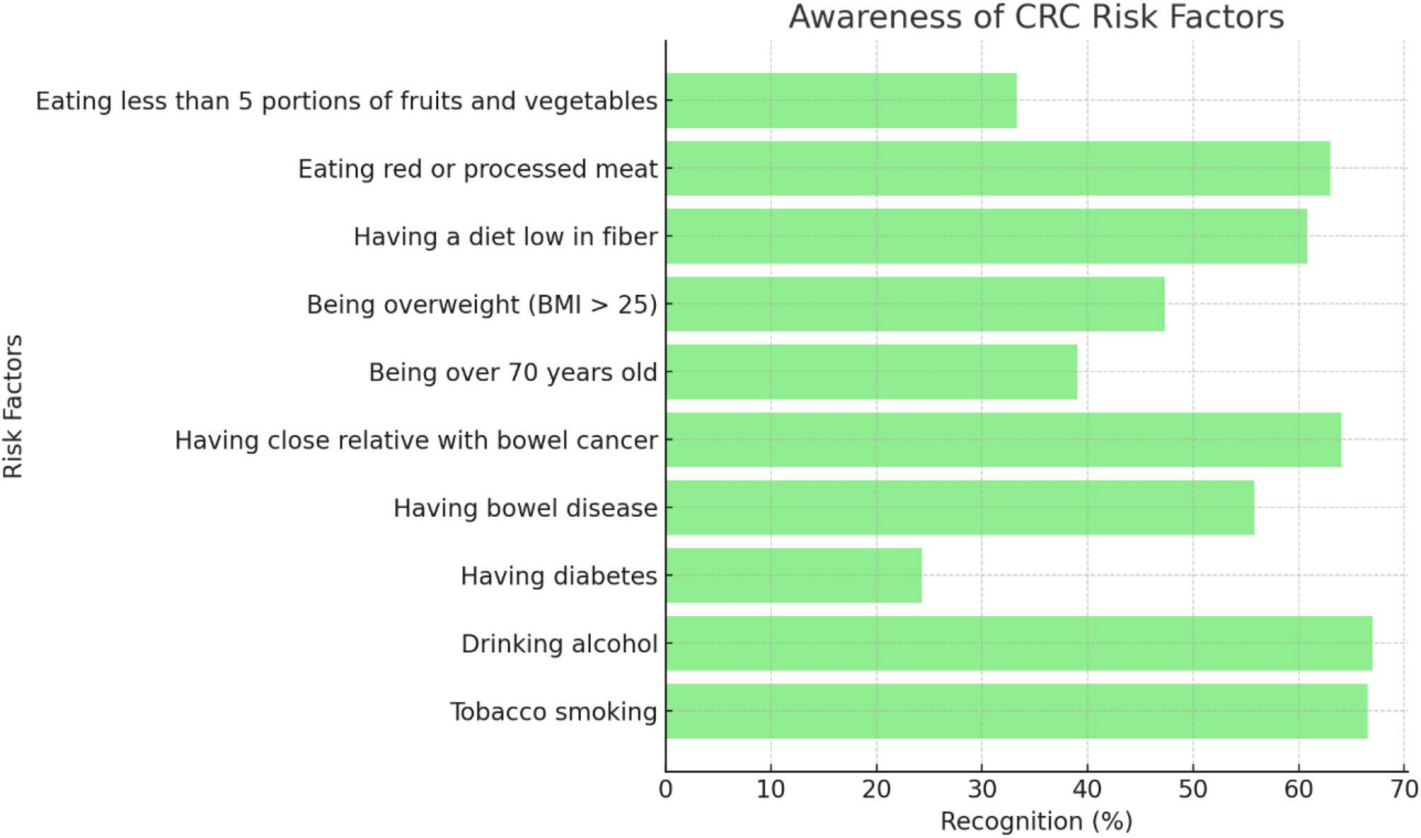
The recognition percentages of CRC risk factors among participants

Table 2 shows the percentages of participants who identified the different symptoms and risk factors of CRC. Out of nine symptoms related to CRC, the most commonly recognized symptoms were ‘lump in abdomen’ (68%), ‘unexplained weight loss’ (67.5%) and ‘bleeding from the back passage’ (66.8%). On the other hand, the least commonly recognized symptoms were ‘pain in back passage’ (37.3%) and ‘bowel does not empty’ (34.5%) (Fig. 2). Furthermore, the most commonly recognized CRC risk factors were ‘drinking alcohol’ (67%), ‘tobacco use’ (66.5%) and ‘Having close relative with bowel cancer’ (64%). ‘diabetes’ (24.3%) and ‘older age’ (39%) were less commonly recognized by the participants as risk factors (Fig. 3).

**Table 2:** Frequency distribution of awareness of CRC symptoms and risk factors among participants (*N* = 400)

Variable	Frequency (*n*)	Percentage (%)	
CRC symptoms			
Bleeding from the back passage	267		66.8
Persistent pain in the abdomen (tummy)	211		52.8
Change in bowel habits (diarrhea or constipation or both) over a period of weeks	232		58.0
Feeling that the bowel does not completely empty after using the lavatory	138		34.5
Blood in the stool	259		64.8
Pain in the back passage	149		37.3
A lump in the abdomen (tummy)	272		68.0
Tiredness/ anemia	189		47.3
Unexplained weight loss	270		67.5
CRC risk factors			
Eating less than 5 portions of fruits and vegetables a day	133		33.3
Eating red or processed meat once a day or more	252		63.0
Having a diet low in fiber	243		60.8
Being overweight (BMI over 25)	189		47.3
Being over 70 years old	156		39.0
Having close relative with bowel cancer	256		64.0
Having bowel disease (e.g., ulcerative colitis, Crohn’s disease)	223		55.8
Having diabetes	97		24.3
Drinking alcohol	268		67.0
Tobacco smoking	266		66.5

• CRC: Colorectal Cancer

• BMI: Body Mass Index (BMI (kg/m²) = Weight (kg) / [Height (m)]²)d

In general, Females were more likely to recognize the link between unhealthy lifestyle behaviors and CRC than their male counterparts as shown in Table 3. Females were more aware about the association of CRC with the following factors: Having close relative with bowel cancer (72.1% vs. 53.4%; *p* < 0.001), tobacco use (69.9% vs. 62.1%; *p* = 0.008), and drinking alcohol (73% vs. 59.2%; *p* < 0.001). However, male participants were more able to identify the association between Body Mass Index (BMI) (54.0% vs. 42.0%; *p* = 0.042) and age (47.1% vs. 32.7%; *p* = 0.01) on the development of CRC.

**Table 3:** The relationship between sex and awareness of lifestyle risk factors for CRC (*N* = 400)

	Male		Female		
CRC risk factors	Frequency	Percentage	Frequency	Percentage	*p*-value
Eating less than 5 portions of fruits and vegetables a day	48	27.6%	85	37.6%	0.065
Eating red or processed meat once a day or more	107	61.5%	145	64.2%	0.483
Having a diet low in fiber	100	57.5%	143	63.3%	0.279
Being overweight (BMI over 25)	94	54.0%	95	42.0%	0.042*
Being over 70 years old	82	47.1%	74	32.7%	0.01*
Having close relative with bowel cancer	93	53.4%	163	72.1%	< 0.001 **
Having bowel disease (e.g., ulcerative colitis, Crohn’s disease)	89	51.1%	134	59.3%	0.187
Having diabetes	49	28.2%	48	21.2%	0.057
Drinking alcohol	103	59.2%	165	73.0%	< 0.001 **
Tobacco smoking	108	62.1%	158	69.9%	0.008*

* Significant p-value < 0.05

** Significant p-value < 0.001

• CRC: Colorectal Cancer

• BMI: Body Mass Index

### The relationship between background characteristics and CRC awareness mean score

Table 4 describes the relationship between the participants’ background characteristics and their CRC awareness mean score. On bivariate analyses, the respondents’ sex was significantly associated with the awareness regarding CRC symptoms. In addition, the participants’ educational level was significantly associated with the awareness of CRC risk factors.

**Table 4:** The association between background characteristics and awareness regarding CRC symptoms and risk factors (*N* = 400)

Variable	Group	Mean Score (Symptoms)	95% CI Lower (Symptoms)	95% CI Upper (Symptoms)	*p*-value (Symptoms)	Mean Score (Risk Factors)	95% CI Lower (Risk Factors)	95% CI Upper (Risk Factors)	*p*-value (Risk Factors)
Age					0.678				0.713
	50–59	4.9299	4.6172	5.2426		5.2399	4.9337	5.5460	
	60 or more	5.0465	4.5846	5.5084		5.1395	4.6993	5.5798	
Sex					0.013*				0.190
	Female	5.2522	4.9070	5.5974		5.3540	5.0138	5.6942	
	Male	4.5977	4.2129	4.9825		5.0172	4.6467	5.3878	
Nationality					0.866				0.395
	Jordanian	4.9716	4.7102	5.2330		5.2274	4.9727	5.4820	
	Non-Jordanian	4.8462	3.0527	6.6396		4.6154	3.0839	6.1469	
Marital Status					0.907				0.918
	Married	4.9738	4.6969	5.2507		5.2128	4.9525	5.4732	
	Single	4.9298	4.2017	5.6580		5.1754	4.3574	5.9934	
Educational Level					0.47				0.047*
	Pre-secondary	5.0000	4.1403	5.8597		5.8571	5.0882	6.6261	
	Secondary	4.6588	4.1668	5.1509		5.4941	5.0145	5.9737	
	University	5.0602	4.7395	5.3808		4.9962	4.6826	5.3099	
Occupational status					0.373				0.770
	Employed	5.2921	4.7624	5.8218		5.1573	4.6483	5.6663	
	Retired	4.7500	4.3719	5.1281		5.1793	4.8177	5.5410	
	Self-employed	4.8182	3.8412	5.7951		4.9091	4.0035	5.8147	
	Unemployed	5.1383	4.5807	5.6959		5.4149	4.8423	5.9875	

* Significant *p*-value < 0.05

### Predictors of CRC awareness

The adjusted linear regression model showed some interesting insights about factors affecting awareness of CRC (Table 5). Age did not show significant differences between groups, with participants aged 60 or older having slightly lower mean scores than those aged 50–59. This difference remained not significant even after adjustment (*p* = 0.628). Sex, however, was a significant factor, with females scoring higher on average than males. This difference remained significant after adjustment, indicating that sex may play a meaningful role in these outcomes.

**Table 5:** The predictors of overall awareness of colorectal cancer among the study participants (linear regression) (*N* = 400)

	Univariate linear analysis			Multiple linear regression	
Variable	Mean (SD)	Unadjusted Difference in mean (95% CI)	*P*-value	Adjusted Difference in mean (95% CI)	*P*-value
Age (years)					
50–59	9.44 (5.50)	Reference		Reference	
60 or more	8.50 (5.54)	0.402 (-0.533, 1.34)	0.399	-0.258 (-1.302, 0.787)	0.628
Sex					
Male	8.31 (5.80)	Reference		Reference	
Female	9.62 (5.19)	0.991 (0.0772, 1.91)	0.034*	1.170 (0.104, 2.235)	0.031*
Nationality					
Jordanian	9.00 (5.40)	Reference		Reference	
Non-Jordanian	9.73 (5.60)	0.737 (-1.83, 3.31)	0.573	-0.893 (-3.516, 1.730)	0.504
Marital Status					
Married	9.08 (5.63)	Reference		Reference	
Single	8.73 (4.84)	0.0813 (-1.22, 1.39)	0.902	-0.545 (-1.912, 0.821)	0.433
Level of Education					
Pre-secondary	10.9 (5.33)	Reference		Reference	
Secondary	10.2 (4.03)	-0.704 (-2.34, 0.930)	0.397	-0.852 (-2.512, 0.807)	0.313
University	10.1 (4.68)	-0.801 (-2.22, 0.616)	0.267	-1.170 (-2.742, 0.401)	0.144
Employment					
Employed	10.45 (4.39)	Reference		Reference	
Retired	9.93 (4.57)	-0.520 (-1.70, 0.657)	0.386	-0.731 (-2.058, 0.595)	0.279
Self-employed	9.73 (4.50)	-0.722 (-2.58, 1.136)	0.445	-0.829 (-2.701, 1.044)	0.385
Unemployed	10.55 (5.02)	0.104 (-1.24, 1.452)	0.880	-0.864 (-2.588, 0.861)	0.325

• R²: 10%

Adjusted R²: 0.0906

* Significant p-value < 0.05

For nationality, no significant differences were observed between Jordanians and non-Jordanians, as the adjusted analysis showed a non-significant difference (*p* = 0.504). Marital status also did not appear to significantly influence scores, with married and single individuals having comparable outcomes in both unadjusted and adjusted analyses (*p* = 0.433). Similarly, education and employment status did not show significant adjusted differences. While individuals with higher education levels and employment generally had higher mean scores, none of these differences reached statistical significance.

### Barriers

**Fig. 4:**
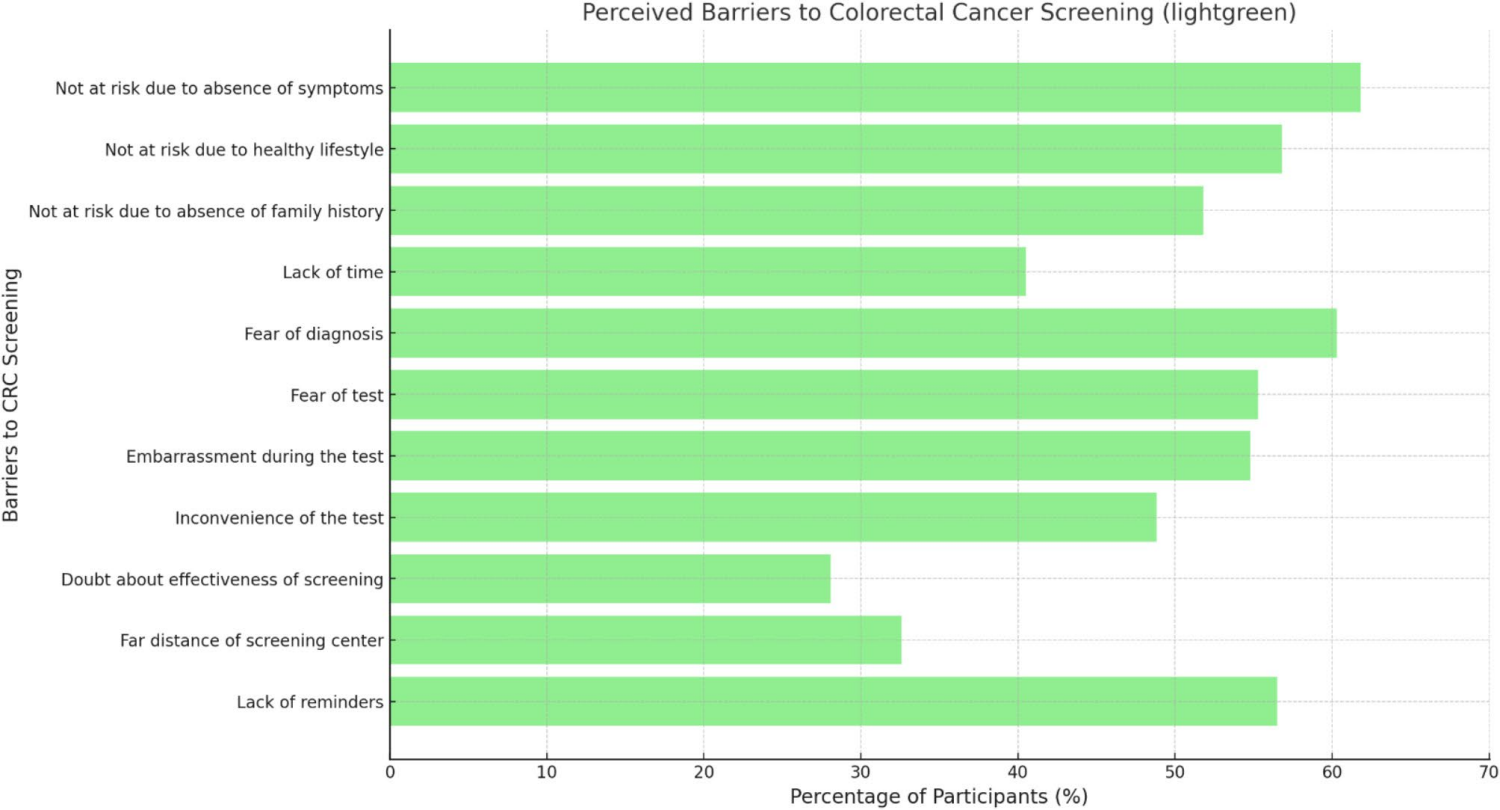
Perceived Barriers to CRC Screening

Table 6 presents variables to assess different barriers for CRC screening. Regarding perceived risk, a significant portion of participants (61.8%) somewhat agreed or strongly agreed (A/SA) that they were unlikely to develop the disease due to an absence of symptoms. Similarly, 56.8% and 51.8% A/SA responses were recorded for a healthy lifestyle and no family history, respectively, being protective factors. Fear of the test (55.3%), embarrassment (54.5%), and inconvenience (48.8) were also identified as test-related barriers. Other barriers included lack of time (41%), lack of reminders (56.5), and fear of diagnosis (60.3%) (Fig. 4).

**Table 6:** Distribution of participants’ responses to barriers for CRC screening

Variable	Do not know	Strongly disagree	Somewhat disagree	Neutral	Somewhat agree	Strongly agree
Not at risk due to absence of symptoms	2.8	7.3	14.8	13.5	36.0	25.8
Not at risk due to healthy lifestyle	2.3	9.0	16.3	15.8	36.5	20.3
Not at risk due to absence of family history	4.5	7.0	19.0	17.8	32.5	19.3
Lack of time	2.3	14.0	22.3	20.5	26.5	14.5
Fear of diagnosis	2.3	10.3	15.0	12.3	30.5	29.8
Fear of test	3.0	8.0	19.3	14.5	29.5	25.8
Embarrassment during the test	3.3	9.8	17.5	15.0	33.5	21.0
Inconvenience of the test	11.3	6.5	12.0	21.5	27.5	21.3
Doubt about the effectiveness of screening	5.3	17.5	29.0	20.3	20.8	7.3
The far distance of the screening center	5.0	15.3	27.0	20.3	20.8	11.8
Lack of reminders	2.0	8.8	14.8	18.0	32.5	24.0

### Barriers by sex

Table 7 reveals some key differences between men and women when it comes to perceived barriers to CRC screening. Females were more likely to believe that a healthy lifestyle reduces their risk of colorectal cancer (62.83% vs. 48.85%, *p* = 0.005). They were also more likely to express fear of the diagnosis (66.37% vs. 52.3%, *p* = 0.004) and fear of the screening test itself (62.83% vs. 45.4%, *p* < 0.001). Embarrassment during the test is also a bigger concern for women (61.95% vs. 44.83%, *p* < 0.001). On the other hand, barriers like lack of reminders or the distance to the screening center seem to affect men and women similarly.

**Table 7:** Sex differences regarding the perceived barriers to undergoing CRC screening (*N* = 400)

Barrier	Female (%)	Male (%)	*P*-value
Not a risk due to absence of symptoms	64.16%	58.62%	0.258
Not a risk due to healthy lifestyle	62.83%	48.85%	0.005*
Not a risk due to absence of family history	54.42%	48.28%	0.222
Lack of time	42.48%	39.08%	0.493
Fear of diagnosis	66.37%	52.3%	0.004*
Fear of test	62.83%	45.4%	< 0.001**
Embarrassment during the test	61.95%	44.83%	< 0.001**
Inconvenience of the test	58.85%	35.63%	< 0.001**
Doubt about the effectiveness of screening	30.97%	24.14%	0.131
The far distance of the screening center	34.96%	29.31%	0.232
Lack of reminders	56.19%	56.9%	0.888

The table includes participants who selected “Strongly Agree” or “Somewhat Agree” for each perceived barrier

* Significant *p*-value < 0.05

** Significant *p*-value < 0.001

### Barriers by educational level

Table 8 shows how educational level influences the perceived barriers to CRC screening. While university-educated participants were slightly more likely to think that factors like the absence of symptoms, a healthy lifestyle, or no family history made screening unnecessary, these differences weren’t statistically significant. What stands out is the concern about the distance to screening centers, which was significantly higher among those with lower education levels. Over half of the pre-secondary group saw distance as a barrier (51.02%), compared to 37.65% of those with secondary education and only 27.44% of university-educated individuals (*p* = 0.003). Other barriers, like lack of time or fear of diagnosis, did not differ much across the groups.

**Table 8:** Educational-level differences regarding the perceived barriers to undergoing CRC screening (*N* = 400)

Barrier	Pre-secondary	Secondary	University Educated	*P*-value
Not a risk due to absence of symptoms	48.98%	55.29%	66.17%	0.29
Not a risk due to healthy lifestyle	44.9%	52.94%	60.15%	0.102
Not a risk due to absence of family history	40.82%	48.24%	54.89%	0.148
Lack of time	28.57%	40.0%	43.61%	0.141
Fear of diagnosis	59.18%	58.82%	60.9%	0.931
Fear of test	42.86%	57.65%	56.77%	0.175
Embarrassment during the test	51.02%	48.24%	57.14%	0.311
Inconvenience of the test	38.78%	42.35%	52.63%	0.084
Doubt about the effectiveness of screening	24.49%	36.47%	25.94%	0.143
The far distance of the screening center	51.02%	37.65%	27.44%	0.003*
Lack of reminders	53.06%	69.41%	53.01%	0.26

The table includes participants who selected “Strongly Agree” or “Somewhat Agree” for each perceived barrier

* Significant *p*-value < 0.05

## Discussion

Colorectal cancer is a growing public health concern worldwide, with incidence and mortality rates continuing to rise [18]. As CRC may remain asymptomatic until advanced stages, raising awareness of its risk factors and symptoms is vital for early detection. Understanding the barriers to CRC screening is also essential, as these obstacles prevent individuals from participating in life-saving preventive measures. In the current study, we aimed to assess the awareness of CRC symptoms and risk factors among adults aged 50–75 years in Jordan. We also aimed to identify the barriers among the same population toward CRC screening.

The overall mean awareness score was 10.18/19 (SD = 2.65), which shows a relatively low level of awareness regarding CRC. These results are in align with previous studies in Jordan, which raised similar concerns. This issue has been evident in nearby countries as well. A study in Lebanon showed that only 31.5% of people knew about CRC symptoms, and just 17.2% were aware of its risk factors [19]. Similarly, a study in Bahrain reported low CRC awareness, with overall knowledge at 56% and specific awareness of symptoms and risk factors at 59% and 53%, respectively [20]. In addition, a recent study in Saudi Arabia recorded a low mean awareness score of 11.05 out of 23 among 5,720 participants [21]. Likewise, a study in Egypt found that awareness of CRC symptoms was low among the Egyptian population with only 29% of participants recognized key CRC risk factors [22]. Supporting these findings, a study from North-Eastern Iran found that the average knowledge score of CRC risk factors was only 3.63/10 [23].

Comparing our results with studies conducted among the same age group (50–75 years), awareness levels appeared to vary across different populations. A study in Qatar found that the mean awareness score of symptoms was 3.63/9, and risk factors awareness was 5.43/11, with an overall awareness score of 9.03/20 [24]. Our study reported higher symptom awareness (4.97/9) but similar risk factor awareness (5.21/10). In contrast, studies outside the Middle East have reported higher CRC awareness levels. A study among Asian Americans reported a mean CRC knowledge score of 6.10/9 [25]. Also, results from Turkey showed higher mean CRC risk factors awareness score (7.3/10) compared to our study [26]. In addition, a study in South Carolina, reported a mean CRC knowledge score of 9.6 out of 14 [27]. These findings, despite differences in scoring scales, suggest higher awareness compared to our study’s overall awareness score of 10.18/19.

The Qatar study, which used a scoring system similar to the one used in our study, revealed that “lump in abdomen” was the most common reported symptom with a percentage of 56.5%, while 71.7% of the participants recognized “daily consumption of processed meat” as the most significant risk factor. The least recognized symptom was “pain in the back passage” (22.5%), a symptom also frequently overlooked by participants in our study [24]. In contrast, a survey conducted in the United Kingdom using the Bowel/Colorectal CAM instrument reported that “blood in stools” was the most recognized symptom (88.6%), and the main identified risk factor was having a “close relative with CRC” (65%) [28]. Both the British study and our research highlighted that “bowel does not empty” was the least known symptom, with awareness levels at 47% and 34.5%, respectively. Notably, “diabetes” was identified as one of the least recognized risk factors across all three studies, with only quarter of participants in our study reporting it as a risk factor. Despite being a well-known risk factor for CRC, the public knowledge about the relation between CRC and diabetes remains limited, as it is often obscured by other known risk factors like family history [29].

Globally, numerous studies have investigated the level of CRC awareness in different communities, with variable results being reported. Consistent with previous studies from Iran, the United Arab Emirates, Hungary, and the United Kingdom, our study also found a low awareness of CRC risk factors [30, 31, 32, 33]. However, other populations reported a higher level of awareness regarding CRC [34, 35, 36]. This discrepancy might be due to differences in study settings and participant characteristics, as our study focused on individuals aged 50 to 75.

Regarding CRC risk factors, there are both modifiable and non-modifiable factors that play a role in developing the disease. Our study found that alcohol consumption was the most recognized modifiable risk factor with 67% of the participants acknowledging its role. This represents well-established awareness regarding the effects of alcohol on cancer development when compared to populations of western countries [36, 37]. The most noted non-modifiable risk factor in our study was having a family member with CRC, with a percentage of 64%. In Palestine however, people seemed to focus more on personal health history like inflammatory bowel disease (IBD) rather than on genetic factors such as family history of CRC [38].

Several key barriers to CRC screening emerged from our study, including no visible symptoms, lack of family history, a belief in a healthy lifestyle, fear of diagnosis, embarrassment, and concerns about the screening procedure. A recent study in Jordan identified that lack of information about screening, fear of any potential complications due to the test, embarrassment associated with colonoscopy, and fear of the result, were considered major barriers to CRC screening among the population [11]. similar barriers were identified in Saudi Arabia, where fear of the procedure itself and of the test results were common obstacles [39]. In Iran, a systematic review identified that cost, shame, fear of cancer diagnosis, and lack of testing recommendation by the physician were the most common barriers to screening, which supports the finding that fear of diagnosis is considered one of the most important barriers to screening [40]. Another cross-sectional study in the United States, which involved adults aged 50 and above, showed that fear of the test, lack of transportation, and financial issues were significant barriers to screening [41].

Interestingly, participants with a pre-secondary level of education showed slightly higher awareness scores in regard to barriers to CRC screening than those with higher education, though not statistically significant. This highlights the need for targeted programs to address gaps at all education levels. This is in contrast to a national cross-sectional study conducted in Palestine, which showed that education below secondary school is considered a barrier to CRC screening [42].

In our study, over half of the participants (51.8%) did not see themselves at risk due to the absence of a family history of CRC, which goes along with similar findings in Lebanon (52%) and Qatar (55.1%) [17, 19]. This highlights a significant lack of awareness, as CRC frequently occurs in individuals without a family history. In addition to that, 61.8% of our participants thought they weren’t at risk for CRC because they lacked symptoms, a belief that was also prevalent in Qatar (60.6%) [17]. A recent study in Jordan, with a sample of 921 individuals of the same age group to ours, reported that‘’feeling well’’ was the most recorded barrier with a percentage of 53.9%, which aligns with our finding [9]. Another study in Saudi Arabia reinforced this finding, as they found that the absence of symptoms was considered a major barrier by 73.4% of participants, highlighting how this misconception is widespread [43]. Additionally, a meta-analysis in 2016 supported this and concluded that the perception that screening is needed only when symptoms appear is considered one of the most important barriers to screening [44]. This misconception may come from the idea that people only need medical care when they feel sick or unwell, rather than for prevention. Also, there are not enough awareness campaigns explaining that CRC can develop initially without symptoms.

Our findings also highlight notable gender differences in screening reluctance. Women were significantly more likely than men to express fear (62.83% vs. 45.4%), embarrassment (61.95% vs. 44.83%), and concerns about discomfort as reasons for avoiding screening. This pattern is consistent with studies from Korea and Iran, which found that women experienced higher levels of anxiety about CRC screening procedures [45, 46].

As noted, findings from different studies indicate that there is in general a low level of awareness regarding CRC and screening programs. However, significant regional differences in awareness are apparent. Public health initiatives should consider these variations when designing targeted educational campaigns, ensuring that the messages are tailored to different populations’ specific needs and perceptions. While our findings are specific to Jordan’s cultural and healthcare context, they can offer valuable guidance for similar settings, especially in the Middle East, where CRC screening faces common challenges.

Our results highlight the need for targeted interventions to improve CRC awareness and screening in Jordan. We recommend that educational campaigns focus on poorly recognized symptoms, such as “pain in the back passage”, and underappreciated risk factors like older age and diabetes. To enhance effectiveness, such campaigns should be tailored to different age groups. In addition to that, sex-specific measurements must be taken into consideration, particularly among women, who reported fear and embarrassment as key barriers. This can be supported by the availability of female healthcare providers in screening programs. Also, Healthcare providers should promote CRC screening during routine visits, addressing common fears and misinformation. To improve accessibility, mobile screening units and localized facilities should be implemented to overcome logistical barriers. Finally, Mass media campaigns including television and social media should spread culturally appropriate messages that highlight the benefits of asymptomatic screening and clarify that CRC screening is a preventive measure, not just a diagnostic tool, which can address the misconception among people that CRC should always be accompanied by symptoms. All of these actions aim to enhance awareness and screening uptake, reducing CRC-related mortality.

Our study has strengths and limitations. First, it is among the few studies to assess colorectal cancer awareness using a validated questionnaire in Jordan. The relatively large sample enhances our results and make it more reliable. Additionally, the study provides valuable insights to help guide the improvement of public health strategies. However, there are some limitations. The cross-sectional design prevents us from establishing relationships between awareness levels and demographic characteristics, and does not allow to track changes over time. Also, the study used a non-probability sampling method, which may limit the generalizability of the findings, as it might not have captured the full diversity of the population, particularly people in rural areas or those from less advantaged backgrounds. This was evident in the percentage of university-educated participants which reached over 65%, compared to less than 15% according to national statistics in Jordan. Additionally, the use of self-reported data may introduce potential bias, as participants might not accurately report their knowledge. Lastly, our study age group (50–75) have lower access to the internet, so an online survey may exclude those with limited internet access and digital devices, illiterate individuals, or those who have health issues such as vision problems. This may lead to underrepresentation of less socioeconomically and less educated individuals.

## Conclusion

Although there has been progress in increasing awareness of CRC, significant gaps remain. Symptoms such as pain in the back passage and a feeling that the bowel does not empty are still poorly recognized, and awareness of key risk factors like chronic conditions and older age is limited. In order to address these gaps, more educational programs and greater involvement from healthcare providers are needed in order to raise awareness and overcome major barriers to CRC screening like fear or lack of knowledge. By applying these measurements, we can significantly improve awareness, increase screening participation, and ultimately reduce the incidence and mortality of CRC.

## Electronic supplementary material

Below is the link to the electronic supplementary material.

Supplementary Material 1

## Data Availability

The datasets used and/or analysed during the current study are available from the corresponding author on reasonable request.

## Abbreviations

CRC: Colorectal Cancer
USPSTF: United States Preventive Services Task Force
BMI: Body Mass Index

## References

[1] Bray F, Laversanne M, Sung H, Ferlay J, Siegel RL, Soerjomataram I, Jemal A. Global cancer statistics 2022: GLOBOCAN estimates of incidence and mortality worldwide for 36 cancers in 185 countries. CA Cancer J Clin. 2024;74:229–63. 38572751 10.3322/caac.21834

[2] Siegel RL, Wagle NS, Cercek A, Smith RA, Jemal A. Colorectal cancer statistics, 2023. CA Cancer J Clin. 2023;73(3). 10.3322/caac.21772 36856579

[3] Morgan E, Arnold M, Gini A, Lorenzoni V, Cabasag CJ, Laversanne M et al. Global burden of colorectal cancer in 2020 and 2040: incidence and mortality estimates from GLOBOCAN. Gut. 2023;72(2). 10.1136/gutjnl-2022-327736 36604116

[4] Nimri O, Arqoup K. Statistical Digest Jordan Cancer Registry (JCR) Cancer Incidence in Jordan– 2016 Non-Communicable Diseases Directorate-MOH. 2020;21.

[5] Davidson KW, Barry MJ, Mangione CM, Cabana M, Caughey AB, Davis EM, et al. Screening for colorectal cancer: US preventive services task force recommendation statement. JAMA. 2021;325(19):1965–77. 34003218 10.1001/jama.2021.6238

[6] Brenner H, Chen C. The colorectal cancer epidemic: challenges and opportunities for primary, secondary and tertiary prevention. Br J Cancer. 2018;119(7):785–92. 30287914 10.1038/s41416-018-0264-x PMC6189126

[7] Sano Y, Byeon JS, Li XB, Wong MCS, Chiu HM, Rerknimitr R, et al. Colorectal cancer screening of the general population in East Asia. Dig Endosc. 2016;28(3):243–9. 26595883 10.1111/den.12579

[8] Sawicki T, Ruszkowska M, Danielewicz A, Niedźwiedzka E, Arłukowicz T, Przybyłowicz KE. A review of colorectal cancer in terms of epidemiology, risk factors, development, symptoms and diagnosis. Cancers (Basel). 2021;13(9):2025. 33922197 10.3390/cancers13092025 PMC8122718

[9] Jadallah K, Khatatbeh M, Mazahreh T, Sweidan A, Ghareeb R, Tawalbeh A, et al. Colorectal cancer screening barriers and facilitators among Jordanians: A cross-sectional study. Prev Med Rep. 2023;32:102149. 36852311 10.1016/j.pmedr.2023.102149 PMC9958352

[10] Taha H, Al Jaghbeer M, Al-Sabbagh MQ, Al Omari L, Berggren V. Knowledge and practices of colorectal cancer early detection examinations in Jordan: A cross sectional study. Asian Pac J Cancer Prev. 2019;20(3):831–8. 30912401 10.31557/APJCP.2019.20.3.831 PMC6825773

[11] Abdulelah ZA, Abdulelah AA, Alqaisieh M, Khanfar AN, Hammad NH, Masoud EB, Al, et al. National survey of barriers to colorectal cancer screening in Jordan. East Mediterr Health J. 2024;30(2):125–35. 38491898 10.26719/emhj.24.028

[12] Goldman RE, Diaz JA, Kim I. Perspectives of colorectal cancer risk and screening among Dominicans and Puerto Ricans: stigma and misperceptions. Qual Health Res. 2009;19(11):1559–68. 19776255 10.1177/1049732309349359 PMC3584335

[13] Unger-Saldaña K, Saldaña-Tellez M, Potter MB, Van Loon K, Allen-Leigh B, Lajous M. Barriers and facilitators for colorectal cancer screening in a low-income urban community in Mexico City. Implement Sci Commun. 2020;1(1):64. 32885219 10.1186/s43058-020-00055-z PMC7427948

[14] Gupta S, Sussman DA, Doubeni CA, Anderson DS, Day L, Deshpande AR, et al. Challenges and possible solutions to colorectal cancer screening for the underserved. J Natl Cancer Inst. 2014;106(4):dju032. 24681602 10.1093/jnci/dju032 PMC3982886

[15] Raosoft. Sample size calculator [Internet]. 2016 [Cited 2024 Oct 6]. Available from: http://www.raosoft.com/samplesize.html

[16] Cancer Research UK. The Cancer Awareness Measures (CAM) [Internet]. 2023 [Cited 2024 Oct 6]. Available from: https://www.cancerresearchuk.org/health-professional/awareness-and-prevention/the-cancer-awareness-measures-cam#CAM_Use0

[17] Al-Dahshan A, Abushaikha S, Chehab M, Bala M, Kehyayan V, Omer M, et al. Perceived barriers to colorectal Cancer screening among eligible adults in Qatar and the associated factors: A cross sectional study. Asian Pac J Cancer Prev. 2021;22(1):45–51. 33507678 10.31557/apjcp.2021.22.1.45 PMC8184193

[18] Rawla P, Sunkara T, Barsouk A. Epidemiology of colorectal cancer: incidence, mortality, survival, and risk factors. Prz Gastroenterol. 2019;14(2):89–103. 31616522 10.5114/pg.2018.81072 PMC6791134

[19] Tfaily MA, Naamani D, Kassir A, Sleiman S, Ouattara M, Moacdieh MP, et al. Awareness of colorectal cancer and attitudes towards its screening guidelines in Lebanon. Ann Glob Health. 2019;85(1):75. 31148437 10.5334/aogh.2437 PMC6634322

[20] Nasaif HA, Al Qallaf SM. Knowledge of colorectal cancer symptoms and risk factors in the Kingdom of Bahrain: A cross- sectional study. Asian Pac J Cancer Prev. 2018;19(8):2299–304. 30139241 10.22034/APJCP.2018.19.8.2299 PMC6171415

[21] Almadi M, Alghamdi F. The gap between knowledge and undergoing colorectal cancer screening using the health belief model: A National survey. Saudi J Gastroenterol. 2019;25(1):27–39. 30618441 10.4103/sjg.SJG_455_18 PMC6373220

[22] Yehia SA, Alboraie M, Ashour R, Hassan D, Ezzat R, El-Raey F, et al. Enhancing colorectal cancer prevention: a National assessment of public awareness in Egypt. BMC Public Health. 2024;24(1):1415. 38802842 10.1186/s12889-024-18746-w PMC11129470

[23] Olyani S, Ebrahimipour H, Mahdizadeh Taraghdari M, Jamali J, Peyman N. Colorectal Cancer awareness and related factors among adults attending primary healthcare in North-Eastern of Iran: A Cross-sectional study. J Res Health Sci. 2023;23(3):e00589. 38315904 10.34172/jrhs.2023.124 PMC10660502

[24] Al-Dahshan A, Chehab M, Bala M, Omer M, AlMohamed O, Al-Kubaisi N, et al. Colorectal cancer awareness and its predictors among adults aged 50–74 years attending primary healthcare in the state of Qatar: a cross-sectional study. BMJ Open. 2020;10(7):e035651. 32641359 10.1136/bmjopen-2019-035651 PMC7342467

[25] Juon HS, Guo J, Kim J, Lee S. Predictors of colorectal Cancer knowledge and screening among Asian Americans aged 50–75 years old. J Racial Ethn Health Disparities. 2017;5(3):545. 28664503 10.1007/s40615-017-0398-1 PMC5762422

[26] Korkmaz M, Jarrar M, Dioso R, Avci İ, Albaker W, Mohamed R, et al. Health beliefs towards colorectal Cancer and associated factors in a three Muslim countries (Turkey, Malaysia, and Saudi Arabia): A screening study of men aged between 50–75. Asian Pac J Cancer Prev. 2025;26(2):515–24. 40022696 10.31557/APJCP.2025.26.2.515 PMC12118013

[27] Brandt HM, Dolinger HR, Sharpe PA, Hardin JW, Berger FG. Relationship of colorectal cancer awareness and knowledge with colorectal cancer screening. Colorectal Cancer. 2012;1(5):383. 26257828 10.2217/crc.12.45 PMC4529290

[28] Power E, Simon A, Juszczyk D, Hiom S, Wardle J. Assessing awareness of colorectal cancer symptoms: measure development and results from a population survey in the UK. BMC Cancer. 2011;11:366. 21859500 10.1186/1471-2407-11-366 PMC3188511

[29] Yu GH, Li SF, Wei R, Jiang Z. Diabetes and colorectal Cancer risk: clinical and therapeutic implications. J Diabetes Res. 2022;2022:1747326. 35296101 10.1155/2022/1747326 PMC8920658

[30] Bidouei F, Abdolhosseini S, Jafarzadeh N, Izanloo A, Ghaffarzadehgan K, Abdolhosseini A, et al. Knowledge and perception toward colorectal cancer screening in East of Iran. Int J Health Policy Manag. 2014;3(1):11–5. 24987716 10.15171/ijhpm.2014.48 PMC4075097

[31] Al-Sharbatti S, Muttappallymyalil J, Sreedharan J, Almosawy Y. Predictors of colorectal cancer knowledge among adults in the united Arab Emirates. Asian Pac J Cancer Prev. 2017;18(9):2355–9. 28950678 10.22034/APJCP.2017.18.9.2355 PMC5720636

[32] Gede N, Reményi Kiss D, Kiss I. Colorectal cancer and screening awareness and sources of information in the Hungarian population. BMC Fam Pract. 2018;19(1):106. 29960585 10.1186/s12875-018-0799-1 PMC6026511

[33] Anderson AS, Caswell S, Macleod M, Craigie AM, Stead M, Steele RJC, et al. Awareness of lifestyle and colorectal Cancer risk: findings from the bewel study. Biomed Res Int. 2015;2015:871613. 26504842 10.1155/2015/871613 PMC4609381

[34] Akduran F, Cinar N. Effects of nursing education on awareness of risk factors for colorectal cancer. Asian Pac J Cancer Prev. 2015;16(14):5763–6. 26320448 10.7314/apjcp.2015.16.14.5763

[35] Knudsen MD, Hoff G, Tidemann-Andersen I, Bodin GE, Øvervold S, Berstad P. Public awareness and perceptions of colorectal Cancer prevention: a Cross-Sectional survey. J Cancer Educ. 2021;36(5):957–64. 32112366 10.1007/s13187-020-01721-5 PMC8520865

[36] Obidike OJ, Rogers CR, Caspi CE. Examining colorectal Cancer risk awareness and food shelf use among health center patients. J Racial Ethn Health Disparities. 2019;6(5):1021–9. 31168698 10.1007/s40615-019-00603-x PMC6739125

[37] Thomsen KL, Christensen ASP, Meyer MKH. Awareness of alcohol as a risk factor for cancer: A population-based cross-sectional study among 3000 Danish men and women. Prev Med Rep. 2020;19:101156. 32685363 10.1016/j.pmedr.2020.101156 PMC7356255

[38] Elshami M, Dwikat MF, Al-Slaibi I, Alser M, Mohamad BM, Isleem WS et al. Awareness of colorectal Cancer risk factors in Palestine: where do we stand?? JCO Glob Oncol. 2022;(8):e2200070. 10.1200/GO.22.00070 PMC9225594 35696626

[39] Al-Hajeili M, Abdulwassi H, Alshadadi F, Alqurashi L, Idriss M, Halawani L. Assessing knowledge on preventive colorectal cancer screening in Saudi Arabia: A cross-sectional study. J Family Med Prim Care. 2019;8(10):3140–6. 31742133 10.4103/jfmpc.jfmpc_508_19 PMC6857381

[40] Mozafar Saadati H, Khodamoradi F, Salehiniya H. Associated factors of survival rate and screening for colorectal Cancer in Iran: a systematic review. J Gastrointest Cancer. 2020;51(2):401–11. 31338727 10.1007/s12029-019-00275-0

[41] Muthukrishnan M, Arnold LD, James AS. Patients’ self-reported barriers to colon cancer screening in federally qualified health center settings. Prev Med Rep. 2019;15:100896. 31193550 10.1016/j.pmedr.2019.100896 PMC6531912

[42] Qumseya BJ, Tayem YI, Dasa OY, Nahhal KW, Abu-Limon IM, Hmidat AM, et al. Barriers to colorectal cancer screening in Palestine: A National study in a medically underserved population. Clin Gastroenterol Hepatol. 2014;12(3):463–9. 24055899 10.1016/j.cgh.2013.08.051

[43] Alduraywish SA, Altamimi LA, Almajed AA, Kokandi BA, Alqahtani RS, Alghaihb SG, et al. Barriers of colorectal cancer screening test among adults in the Saudi population: A Cross-Sectional study. Prev Med Rep. 2020;20:101235. 33194537 10.1016/j.pmedr.2020.101235 PMC7645071

[44] Honein-AbouHaidar GN, Kastner M, Vuong V, Perrier L, Daly C, Rabeneck L, et al. Systematic review and meta-study synthesis of qualitative studies evaluating facilitators and barriers to participation in colorectal cancer screening. Cancer Epidemiol Biomarkers Prev. 2016;25(6):907–17. 27197277 10.1158/1055-9965.EPI-15-0990

[45] Lee SY. Koreans’ awareness and preventive behaviors regarding colorectal Cancer screening. Asian Pac J Cancer Prev. 2018;19(9):2657. 30256565 10.22034/APJCP.2018.19.9.2657 PMC6249469

[46] Majidi A, Majidi S, Salimzadeh S, Khazaee-Pool M, Sadjadi A, Salimzadeh H, et al. Cancer screening awareness and practice in a middle income country; A systematic review from Iran. Asian Pac J Cancer Prev. 2017;18(12):3187. 29281865 10.22034/APJCP.2017.18.12.3187 PMC5980869

