# Supplementary material for: Assessing awareness of colorectal cancer symptoms, risk factors and screening barriers among eligible adults in Jordan: a cross-sectional study
Source: BMC Public Health. 2025 Apr 25;25:1544. doi: 10.1186/s12889-025-22800-6 (PMC12023689; doi:10.1186/s12889-025-22800-6)
Supplement: Supplementary file 1 — Supplementary Material 1 [file 12889_2025_22800_MOESM1_ESM.docx]

|  | **Bowel Cancer Awareness Questionnaire**  **استبيان عن الوعي بسرطان القولون** | | | | | |
| --- | --- | --- | --- | --- | --- | --- |
|  | **Questionnaire no.: :رقم استبيان** | | | | | |
| **Sociodemographic data**  **البيانات الاجتماعية الديموغرافية** | | | | | |  |
| كم عمرك؟  How old are you? | | | | | | 1 |
|  | | | | | |  |
| What is your gender?  ما هو جنسك؟ | | | | | | 2 |
|  | |  | | | Male  ذكر |  |
|  |  |  | | | Female  انثى |  |
| ما هي جنسيتك؟  What is your nationality? | | | | | | 3 |
| Jordanian اردني. | | | | | |  |
| Non-Jordanian غير اردني | | | | | |  |
| What is your marital status?  ما هي حالتك الاجتماعية؟ | | | | | | 4 |
|  | |  | | | متزوج Married |  |
|  |  |  | | | أعزبSingle |  |
|  |  |  |  |  |  |  |
| What is your educational level?  ما هو مؤهلك العلمي؟ | | | | | | 5 |
|  | |  | | | Pre-secondary ما قبل الثانوي. |  |
|  | |  | | | Secondaryثانوي. |  |
|  | |  | | | Universityجامعي. |  |
| What is your current occupational status?  ما هو وضعك الوظيفي الحالي؟ | | | | | | 6 |
|  | |  | | | Employed  موظف |  |
|  |  |  |  |  |  |  |
|  | |  | | | Unemployed  غير موظف |  |
|  |  |  |  |  |  |  |
|  | |  | | | Self-employed  عمل خاص |  |
|  | |  | | | Retired  متقاعد |  |

|  |  |  | **Which of the followings are symptoms for bowel cancer. We are interested in your opinion:**  **أي من الأعراض التالية تعتبر أعراض لسرطان الأمعاء؟**  **نود أن نعرف رأيك** | 7. |
| --- | --- | --- | --- | --- |
| Don't know  لا أعرف | No  لا | Yes  نعم |  |  |
|  |  |  | Do you think bleeding from the back passage could be a sign of bowel cancer?  هل تعتقد أن خروج الدم من فتحة الشرج قد يكون علامة على الإصابة بسرطان الأمعاء؟ | 7.1 |
|  |  |  | Do you think persistent pain in the abdomen (tummy) could be a sign of bowel cancer?  هل تعتقد ان الألم المستمر فً البطن قد يكون علامة على الإصابة بسرطان الأمعاء؟ | 7.2 |
|  |  |  | Do you think a change in bowel habits (diarrhea, constipation or both) over a period of weeks could be a sign of bowel cancer?  هل تعتقد أن التغير في عادة / طبيعة الإخراج )الإسهال،أو الامساك، أو كلاهما معا( لمدة اسابيع قد يكون علامة على الإصابة بسرطان الأمعاء؟ | 7.3 |
|  |  |  | Do you think a feeling that the bowel does not completely empty after using the lavatory could be a sign of bowel cancer?  هل تعتقد أن الشعور بعدم إفراغ الأمعاء بشكل كامل بعد استخدام المرحاض قد يكون علامة على الإصابة بسرطان الأمعاء؟ | 7.4 |
|  |  |  | Do you think blood in the stools could be a sign of bowel cancer?  هل تعتقد أن وجود دم في البراز قد يكون علامة على الإصابة بسرطان الأمعاء؟ | 7.5 |
|  |  |  | Do you think pain in the back passage could be a sign of bowel cancer?  هل تعتقد أن الألم في منطمة الشرج قد يكون علامة على الإصابة بسرطان الأمعاء؟ | 7.6 |
|  |  |  | Do you think a lump in the abdomen (tummy) could be a sign of bowel cancer?  هل تعتقد أن وجود كتله في البطن قد يكون علامة على الإصابة بسرطان الأمعاء؟ | 7.7 |
|  |  |  | Do you think that tiredness/anemia could be a sign of bowel cancer?  هل تعتقد أن التعب / فقر الدم قد يكون علامة على الإصابة بسرطان الأمعاء؟ | 7.8 |
|  |  |  | Do you think unexplained weight loss could be a sign of bowel cancer?  هل تعتقد أن الفقدان غير المبرر للوزن قد يكون علامة على الإصابة بسرطان الأمعاء؟ | 7.9 |

|  |  |  | **Which of the followings are risk factors for bowel cancer? We are interested in your opinion:**  **أي من الحالات التالية تعتبر من عوامل الخطر لسرطان الأمعاء؟**  **نود أن نعرف رأيك:** | | 8 |
| --- | --- | --- | --- | --- | --- |
| Don't know  لا أعرف | No  لا | Yes  نعم |  | |  |
|  |  |  | Eating less than 5 portions of fruits and vegetables a day  تناول اقل من 5 حصص من الفواكه والخضروات في اليوم | | 8.1 |
|  |  |  | Eating red or processed meat once a day or more  تناول اللحوم الحمراء أو المجهزة / المصنعة مرة واحدة في اليوم أو أكثر | | 8.2 |
|  |  |  | Having a diet low in fiber | إتباع نظام غذائي منخفض الالياف | 8.3 |
|  |  |  | Being overweight (BMI over 25)  الوزن الزائد )مؤشر كتلة الجسم أكثر من (25 | | 8.4 |
|  |  |  | Being over 70 years old | أن يكون العمر فوق 70 سنة | 8.5 |
|  |  |  | Having a close relative with bowel cancer  وجود قريب من الدرجة الأولى لديه سرطان القولون | | 8.6 |
|  |  |  | Having a bowel disease (e.g. ulcerative colitis, Crohn’s disease)  الإصابة بمرض في الأمعاء )على سبيل المثال:التهاب القولون التقرحي، أو داء كرون( | | 8.7 |
|  |  |  | Having diabetes | وجود مرض السكري | 8.8 |
|  |  |  | Drinking alcohol | شرب الكحول | 8.9 |
|  |  |  | Tobacco smoking | تدخين التبغ | 8.10 |

|  | | | | | | |  |  |
| --- | --- | --- | --- | --- | --- | --- | --- | --- |
| **To what extent do you think the following factors could form barriers to undertake annual bowel screening?**  **إلى أي مدى تعتقد أن العوامل التالية ممكن أن تمثل عائقا لإجراء الكشف المبكر السنوي لسرطان الأمعاء؟** | | | | | | | | 9 |
| Do not know  لا أعلم | Strongly disagree  لا اوافق  بشدة | Somewhat disagree  لا اوافق الى حد  ما | Neutral  محايد | Somewhat disagree  اوافق إلى حد  ما | Strongly agree  اوافق بشدة |  | |  |
|  |  |  |  |  |  | Not at risk due to absence of symptoms  لست في خطر بسبب عدم وجود أعراض | | 9.1 |
|  |  |  |  |  |  | Not at risk due to healthy lifestyle  لست في خطر بسبب اتباعي لنمط حياة صحي | | 9.2 |
|  |  |  |  |  |  | Not at risk due to absence of family history  لست في خطر بسبب عدم وجود تاريخ مرضي بالعائلة | | 9.3 |
|  |  |  |  |  |  | Lack of time  عدم توفر الوقت | | 9.4 |
|  |  |  |  |  |  | Lack of reminders  عدم وجود تذكير | | 9.5 |
|  |  |  |  |  |  | Fear of diagnosis  الخوف من التشخيص | | 9.6 |
|  |  |  |  |  |  | Fear of test  الخوف من الفحص | | 9.7 |
|  |  |  |  |  |  | Embarrassment during the test  الإحراج أثناء الفحص | | 9.8 |
|  |  |  |  |  |  | Inconvenience of the test  الفحص غير مريح | | 9.9 |
|  |  |  |  |  |  | Doubt about effectiveness of screening  لدي شك حول فاعلية الفحص | | 9.10 |
|  |  |  |  |  |  | The far distance of the screening center  المسافة البعيدة من مركز الفحص | | 9.11 |
